# Survey of various carbapenem-resistant mechanisms of *Acinetobacter baumannii* and *Pseudomonas aeruginosa* isolated from clinical samples in Iran

**DOI:** 10.22038/IJBMS.2020.44853.10463

**Published:** 2020-11

**Authors:** Leila Azimi, Fatemeh Fallah, Abdollah Karimi, Mehdi Shirdoust, Taher Azimi, Iraj Sedighi, Mohammad Rahbar, Shahnaz Armin

**Affiliations:** 1 Pediatric Infections Research Center, Research Institute for Children’s Health, Shahid Beheshti University of Medical Sciences, Tehran, Iran; 2 Department of Pathobiology, School of Public Health, Tehran University of Medical Sciences, Tehran, Iran; 3 Pediatric department, faculty of medicine, Hamadan University of Medical Sciences, Hamadan, Iran; 4 Department of Microbiology, Reference Health Laboratories Research Center, Ministry of Health and Medical Education, Tehran, Iran

**Keywords:** Acinetobacter baumannii, Carbapenems, Drug resistance, Iran, Pseudomonas aeruginosa

## Abstract

**Objective(s):**

*Pseudomonas aeruginosa* and *Acinetobacter baumannii* resist antibiotics by different intrinsic and acquired mechanisms. This study aims to define various carbapenem-resistant mechanisms of isolated *P. aeruginosa* and *A. baumannii* from nine different provinces of Iran.

**Materials and Methods:**

In this cross-sectional study, all carbapenem-resistant *P. aeruginosa* and *A. baumannii* samples from nine provinces of Iran on a one-year time horizon were gathered. Modified Hedge Test (MHT) and Carba NP-Test were applied to the identification of producing-carbapenemase strains. The most important carbapenemase genes recognized by PCR and gene overexpression of the efflux pump were surveyed by efflux pump inhibitors (EPIs) and confirmed by Real-Time PCR.

**Results:**

Twenty-one percent and 43.5% of *P. aeruginosa* and *A. baumannii* isolates were resistant to carbapenem, respectively. MHT and Carba-NP tests identified 21% and 11% carbapenemase-producing strains in these Gram-negative bacteria, respectively. NDM-1 was the most prevalently detected carbapenemase in *P. aeruginosa*; OXA-51 and OXA-23 were the most significant genes in *A. baumannii*. EPIs identified active efflux pumps in 20% and 28% of *P. aeruginosa* and *A. baumannii*, respectively. Real-time PCR confirmed gene overexpression of efflux pumps in 54% and 30% of positive EPIs in *P. aeruginosa* and *A. baumannii*, respectively.

**Conclusion:**

*P. aeruginosa* and *A. baumannii* may become multi-drug-resistant (MDR) and Extensively Drug-Resistant (XDR) strains and cause a high rate of mortality and morbidity. Thus, it is of necessity to prohibit the spread of antibiotic-resistant strains in hospitals.

## Introduction

Carbapenems are broad-spectrum beta-lactam antibiotic agents. They are usually considered the last choice for antibiotic therapy, especially in combatting Extended-Spectrum Beta-Lactamase (ESBL) producing microorganisms (1). Although some alternative antibiotics such as tigecycline and colistin can be used in case of carbapenem resistance, these are characterized by low effectiveness and/or high toxicity (1). The rate of carbapenem resistance to Gram-negative bacteria, especially in nosocomial pathogens such as *P. aeruginosa* and *Acinetobacter baumannii*, is high and increasing steadily (1-3). Nosocomial isolated *P. aeruginosa* and *A. baumannii* may be resistant to most of the available antibiotics and act as Multi-Drug Resistant (MDR) and Extensive Drug-Resistant (XDR) strains (3-5). The presence of carbapenem-resistant bacteria can be quite considerable because they enjoy the chance to shift to MDR strains commonly (1). *P. aeruginosa* and *A. baumannii* can become resistant to carbapenem through various mechanisms (2, 3, 6). The most important mechanism is the potential to produce carbapenemase because most of the carbapenemase genes can be found on the transferable genetic elements and they spread rapidly among bacteria (3, 5, 7). Different classes of carbapenemase can be detected in Gram-negative bacteria including Ambler classes A, B, and D β-lactamases (8). One of the inherent resistant mechanisms of carbapenems is the presence of active efflux pumps. It is important because it can cause cross-resistance to other antibiotic families (2, 9). Resistance to most of the available antibiotics in *P. aeruginosa* and *A. baumannii* can become a complex challenge for physicians due to the limited number of choices left for antibiotic therapy. This study is a multicenter research that aims to evaluate different mechanisms of carbapenem-resistant *P. aeruginosa* (CRPA) and *A. baumannii* (CRAB) through phenotypic and molecular techniques.

## Materials and Methods

***Setting and bacterial isolates***

In this cross-sectional study, *P. aeruginosa* and *A. baumannii* strains were collected from nine provinces of Iran from September 2016 up to September 2017. 

***Antibiotic susceptibility testing***

Carbapenem susceptibility was evaluated according to CLSI guidelines (10). *P. aeruginosa* ATCC 27853 was adopted as the control strain. All Carbapenems-resistant strains were included in the study. 

***Phenotypic screening of carbapenemase-producing strains***

Phenotypic screening of carbapenemase-producing strains was carried out by the Modified Hodge Test (MHT) (10) and CarbaAcineto NP test according to CLSI guidelines (2016)(11). 

***Modified Hodge Test (MHT)***

MHT was accomplished to identify carbapenemase-producing *A. baumannii* by using *E. coli* ATCC 25922 and ertapenem disc (10 µg). Strains with cloverleaf images of inhibition zone were considered as carbapenemase-producing strains according to the CLSI guidelines (2016)(10).

***Carba NP test and CarbaAcineto NP test***

THE CarbaAcineto NP test method has been described previously (11). In brief, one loop of a suspected strain was suspended in Tris-HCL mmol/l (5 M NaCl in CarbaAcineto NP Test) as a lysis buffer from antibiogram plates, vortexed for one min, and then incubated at room temperature for 30 min. The bacterial suspension was centrifuged at 10,000 xg at room temperature for 5 min. Next, 30 µl of the supernatant was mixed in 96 wells with 100 µl of imipenem monohydrate solution (3 mg per ml) pH 7.8, phenol red solution, and 0.1 mmol/l ZnSO_4 _(11).

***Molecular detection of carbapenemase genes***

The most prevalent carbapenemase genes were detected by conventional PCR. These genes included *VIM, IMP, NDM-1, SPM-1, KPC, GES, *and* OXA-48* in *P. aeruginosa* and *A. baumannii *and *OXA-23, OXA-40, OXA-24, OXA-58,* and *OXA-51 *only in *A. baumannii*. Table 1 lists primers and Table 2 shows the previously described PCR conditions (12-18). 

***Phenotypic screening of active efflux pumps***

*Treatment of the efflux pump by inhibitor*

Phenotypic discovery of active efflux pumps was facilitated by detecting Minimum Inhibitory Concentration (MIC) of imipenem ranging between 2-256 µg/ml with and without Cyanide 3-Chlorophenylhydrazone (CCCP) as an EPI. The final concentration of CCCP (C2759 Sigma-Aldrich, France) was 25 µg/ml, simultaneously (19). The positive condition for the presence of active efflux pumps in the isolates was, at least, the 4-fold reduction of MIC in the presence of CCCP. *A. baumannii *ATCC 19606 was used as the control strain.

***Relative gene expression by real-time PCR***

RNA extraction was carried out by the Thermo RNA extraction kit (cat. No. K0732) according to the manual’s instructions. 

We used an RNeasy Mini Kit with 1 hr on-column DNase digestion (Qiagen NV, Venlo, The Netherlands) for purification of total RNA. Total RNA was quantified using a spectrophotometer (WPA Biowave II Nanospectrophotometer, USA) and ratio of absorbance at 260 nm vs 280 nm was used to assess RNA purity. Moreover, extracted RNA was screened on a 3% agarose gel.

At the next step, cDNA synthesis was executed by the Thermo kit (cat. No. K1622). Finally, the gene overexpression of *MexX, MexC, and MexA* in *P. aeruginosa* and of *adeB* in *A. baumannii* from RND-type efflux systems, involved in carbapenem resistance, was prepared. 16srRNA was used as a house-keeping gene and *P. aeruginosa* ATCC 27853 and *A. baumannii* ATCC 19606 were considered as reference strains. The primers are shown in Table 1. Gene overexpression was calculated by the 2^-ΔΔct^ formula (20). 

^a^ Y=T or C; D=A or G or T

^b ^The relative gene expression was calculated for this gene by Real-Time PCR. Corbett Rotor-Gene 6000

***Statistical analysis***

SPSS 22.0 statistical software (IBM Corp., Armonk, USA) was utilized to conduct data analysis. Mean, Confidence Interval (CI), etc. were analyzed by the Explore test in SPSS version 22.0 software. Sensitivity and specificity of phenotypic methods were calculated through the following formula (21):

Sensitivity= (a/(a+c))×100

Specificity= (d/(b+d))×100

Positive predictive value (PPV)= (a/(a+b))×100

Negative predictive value (NPV)=(d/(c+d))×100

## Results

In this cross-sectional study, 675 *P. aeruginosa* and 869 *A. baumannii* remained definite throughout the study, and 140 (20.7%) and 383 (44%) of them, respectively, were resistant to carbapenem. The results of MHT and Carba NP tests used to identify carbapenemase-producing strains are shown in Table 3.

**Figure 1:**
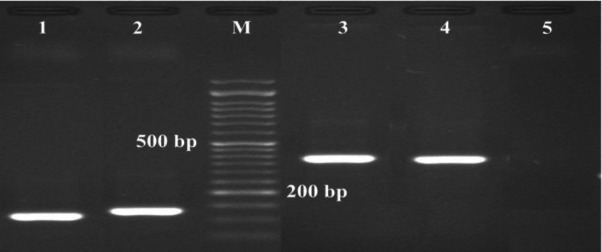
PCR based identification of the NDM-1, imp, vim, and oxa-48 genes, using species primer pairs in *P. aeruginosa* and *A. baumannii* isolates

**Figure 2:**
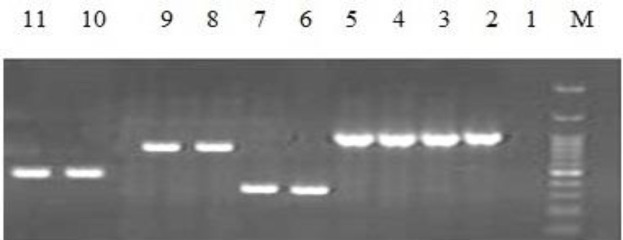
PCR based identification of the oxa-23, oxa-24, oxa-51 and oxa-40 genes, using species primer pairs in P. aeruginosa and A. baumannii isolates

According to the results from molecular detection of carbapenemase by PCR, NDM-1 was the most prevalent enzyme in CRPA and OXA-51 and OXA-23 were the most prevalent genes in CRAB. SPM-1, KPC, GES, and OXA-58 were not observed in any of the strains (Figures 1 and 2) (Tables 4 and 5). 

Sensitivity and specificity of MH and Carba NP tests concerning two non-fermentative Gram-negative bacteria are shown in Table 6. 

**Figure 3:**
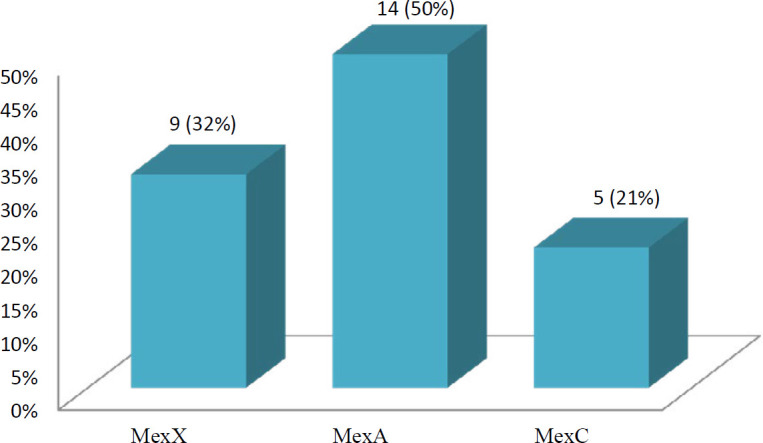
Results of gene expression of each efflux pump in Efflux Pump Inhibitors (EPIs) positive *Pseudomonas aeruginosa*

According to phenotypic evaluations, it was found that 28 (20%) CRPA and 108 (28%) CRAB had active efflux pumps by adding CCCP. In the process of the real-time PCR assay, 15 (54%) *P. aeruginosa *with positive IEPs showed overexpression of MexX, MexC, and MexA (Figure 3). The mean gene expression of *MexX* was 8.34 with CI 95%: 1 to 17.15. The mean gene expression of *MexA* was 67.91 with CI 95%: 16.33 to 119.50. The mean gene expression of *MexC* was 2.73 with CI 95%: 1 to 5.7. As a common efflux pump, *AdeB* gene overexpression was detected in 32 (30%) positive EPI tests of *A. baumannii* with a mean gene expression of *AdeB* leveled at 9.81, CI 95%: 3.37 to 16.25.

**Table 1:** Primers used in this study for detection of resistance genes among *P. aeruginosa* and *A. baumannii* isolates

Gene	Primer sequencing 5’ 3’	PCR product size (bp)	Tm (^o^C)	Reference
*kpc*-F	**CTGTCTTGTCTCTCATGGCC**	**636**	**57.98**	**[12]**
*kpc*-R	**CCTCGCTGTGCTTGTCATCC**		**61.36**	
*ges* -F	**GTTTTGCAATGTGCTCAACG**	**371 **	**57.09**	** [13]**
*ges*-R	**TGCCATAGCAATAGGCGTAG**		**57.54**	
*vim* -F	**GATGGTGTTTGGTCGCATA**	**390**	**55.61**	**[14]**
*vim* -R	**CGAATGCGCAGCACCAG**		**59.54**	
*imp* -F	**TTGACACTCCATTTACDG** ^a^	**139**	**48.56**	**[14]**
*imp*-R	**GATYGAGAATTAAGCCACYCT** ^a^		**51.92**	
*NDM-1* -F	**CCCGGCCACACCAGTGACA**	**129**	**64.73**	**[14]**
*NDM-1*-R	**GTAGTGCTCAGTGTCGGCAT**		**60.11**	
*SPM-1*- F	**GGGTGGCTAAGACTATGAAGCC**	**447**	**60.49**	**[14]**
*SPM-1*-R	**GCCGCCGAGCTGAATCGG**		**63.90**	
*oxa-48*-F	**CCAAGCATTTTTACCCGCATCKACC**	**389**	**63.21**	**[15]**
*oxa-48*-R	**GYTTGACCATACGCTGRCTGCG**		**62.30**	
*oxa*-23- F	**GATGTGTCATAGTATTCGTCGT**	**1058**	**55.86**	**[6]**
*oxa*-23- R	**TCACAACAACTAAAAGCACTGT**		**56.69**	
*oxa-40*- F	**GGAATTCCATGAAAAAATTTATACTTCC**	**846**	**56.44**	**[17]**
*oxa-40* - R	**CGGGATCCCGTTAAATGATTCCAAGATTTTCTAGCG**		**68.57**	
*oxa-24*- F	**GGTTAGTTGGCCCCCTTAAA**	**246**	**57.39**	**[18]**
*oxa-24*- R	**AGTTGAGCGAAAAGGGGATT**		**57.41**	
*oxa-58*- F	**AAGTATTGGGGCTTGTGCTG**	**598**	**58.45**	**[18]**
*oxa-58*- R	**CCCCTCTGCGCTCTACATAC**		**59.68**	
*oxa-51*- F	**TAATGCTTTGATCGGCCTTG**	**353**	**56.48**	**[18]**
*oxa-51*- R	**TGGATTGCACTTCATCTTGG**		**56.01**	
^b^ *AdeB* - F	**AACGGACGACCATCTTTGAGTATT**	**84**	**60.32**	**[36]**
^b^ *AdeB *- R	**CAGTTGTTCCATTTCACGCATT**		**58.36**	
^b^1*6srRNA*-F	**CAGCTCGTGTCGTGAGATGT**	**151**	**60.11**	**[37]**
^b^ *16srRNA*-R	**CGTAAGGGCCATGATGACTT**		**57.67**	
^b^ * MexX-F*	**TGAAGGCGGCCCTGGACATCAGC**	**326**	**69.22**	**[2]**
^b^ * MexX-R*	**GATCTGCTCGACGCGGGTCAGCG**		**69.78**	
^b^ * MexA-F*	**CGACCAGGCCGTGAGCAAGCAGC**	**316**	**70. 52**	**[2]**
^b^ * MexA-R*	**GGAGACCTTCGCCGCGTTGTCGC**		**70.42**	
^b^ * MexC-F*	**GTACCGGCGTCATGCAGGGTTC**	**164**	**65.93**	**[2]**
^b^ * MexC-R*	**TTACTGTTGCGGCGCAGGTGACT**		**67.14**	

**Table 2:** PCR conditions used in this study for detection of carbapenem-resistant genes

**Number of cycles**	**Time **	**Temperature (** ^o^ **C)**	**Cycle**
1	1-10^a^ min	94	Initial denaturation
30-40	30- 45^a^ sec	94	Denaturation Annealing Extension
	30- 40^a^ sec	54- 63^a^	
	30 sec-1^a^ min	72	
1	1-7^a^ min	72	Final extension

^a^ based on each gene

**Table 3:** Results of MHT of carbapenem-resistant strains

MHT & Carba NP negative (%)	Carba NP positive (%)	MHT positive (%)	Bacteria
102 (73)	17 (12)	21 (15)	*P. aeruginosa*
258 (67)	38 (10)	87 (23)	*A. baumannii*

MHT: Modified Hodge Test

M: marker 50bp (SMO321, Fermantas). 1: positive NDM-1: 129bp, 2: positive imp: 139bp, 3: positive vim:390bp, 4: positive oxa-48:389 bp, and 5: negative control

M: marker 100bp (SMO321, Fermantas). 1: negative control. 2- 5: positive oxa-23: 1058 bp. 6, 7: positive oxa-24: 246 bp. 8,9: positive oxa-40: 846 bp. 10, 11: positive oxa-51: 353 bp

**Table 4:** Number (%) of detected carbapenemase genes in carbapenem-resistant *Pseudomonas aeruginosa*

OXA_48	GES	KPC	SPM-1	NDM-1	IMP	VIM	
**6 (4)**	**_**	**_**	**_**	10 (7)	8 (6)	_	*P. aeruginosa*

**Table 5:** Number (%) of detected carbapenemase genes in carbapenem-resistant *Acinetobacter baumannii*

OXA-58	OXA-24	OXA-40	OXA-51	OXA-23	OXA_48	GES	KPC	SPM-1	NDM-1	IMP	VIM	
_	252 (65.8%)	85 (22.3%)	383 (91.6%)	290 (76.5%)	59 (15.4%)	**_**	**_**	**_**	36 (9.4%)	1 (0.3%)	7 (2%)	*A. baumannii*

**Table 6:** Evaluation of MH and Carba NP test in detecting carbapenemase

Carba NP test (%)					MHT (%)					
Accuracy	NPV^**^	PPV^*^	Specificity	Sensitivity	Accuracy	NPV^**^	PPV^*^	Specificity	Sensitivity	Bacteria
9	92	23	92	23	76	89	5	83	7	*P. aeruginosa*
16	3	97	89	14	21	3	98	89	20	*A. baumannii*

* Positive predictive value ; ** Negative predictive value; MH: Modified Hedge Test

## Discussion

In recent decades, CRPA and CRAB have been two of the most critical nosocomial pathogens threatening public health and the World Health Organization has included them in a global priority pathogens list of antibiotic-resistant bacteria (2, 4, 5, 7). Rapid horizontal spread of plasmid-borne carbapenemase in these bacteria can be one of the reasons that there is large-scale spread of carbapenem-resistant bacteria. According to the results of the conducted antibiotic susceptibility testing, 21% of the collected *P. aeruginosa* isolates were resistant to carbapenem. A research group from Brazil worked on *P. aeruginosa* isolated from blood (22). Their results confirmed that 44% of the mentioned isolates were resistant to carbapenems (22). The frequency of CRPA in the Brazilian study is higher than that in our results; therefore, the sources of collected bacteria may justify this difference in frequency. Ghasemian *et al.* (2019) published a review article about the frequency of CRPA and analyzed 36 studies from Iran (23). They reported the detection of CRPA in 55% of the studied isolates (22). We had access to materials and methods (the same as those of other studies) at 10 lab centers and different sorts of clinical specimens. Thus, the discrepancy between our proposed results and those in other studies may correspond to different specimens, materials, and methods (22, 23). 

Production of carbapenemase is one of the significantly responsible mechanisms. NDM-1 is the most frequent carbapenemase in CRPA, as confirmed by the results of PCR and sequencing in the current study. NDM-1 is found on the plasmid and can carry other antibiotic-resistant genes (24). Therefore, the presence and identification of NDM-1 positive strains are quite important and the top priority for control by the nosocomial infection committee of each hospital. The results of a published study revealed that NDM-1-producing *P. aeruginosa* was not detected in or reported from Iran (25). Hence, the detection of NDM-1-producing *P. aeruginosa* in the current study is a very alarming sign for the health care system and it needs a significant approach.

Some phenotypic tests have been proposed so far to detect carbapenemase-producing organisms. MHT and Carba NP tests are two challenging methods. In the current study, the sensitivity and specificity of MHT and Carba NP tests to detecting carbapenemase in both of the bacteria under study are low and reasonable, respectively. In addition, other studies reported acceptable specificity but low sensitivity for MHT (26-28). 

Efflux pumps are the other important carbapenem-resistant mechanisms that can cause the appearance of MDR and XDR strains because they can reject a different family of antibiotics, simultaneously (9). In the current study, 20% of CRPA showed active efflux pumps by the EPIs method. The results obtained in other studies showed the role of active efflux pumps in 18% of CRPA by EPIs (2), similarly to our findings. The results of Real-Time PCR confirmed 54% gene overexpression of the Mex family of efflux pumps in EPIs positive CRPA in this study. However, Azimi *et al.* reported gene overexpression in the Mex family of efflux pumps in 100% CRPA with the CCCP positive test (29). They used different methods for detecting the MIC method and EPI, which may explain the dissimilar results.

In the current study, 44% of the collected *A. baumannii* were resistant to at least one member of the carbapenem class. El Kettani A *et al.* (2017) showed that 76% of *A. baumannii* isolated from blood cultures were resistant to carbapenem (30). In 2018, researchers reported that 80% of *A. baumannii* isolated from wound burn specimens were imipenem-resistant (31). In the above two studies, strains were isolated from blood culture (30) and wounds burn (31); however, the current study evaluated the *A. baumannii* isolated from different clinical samples. We believe that the source of collected specimens and the use of different antibiotic discs in the brand (from different companies) can justify the divergence of our results from those in other studies. The concentration of more than one carbapenemase was observed in 1% and 2% of *P. aeruginosa* and *A. baumannii, *respectively. In addition, gene overexpression of the efflux pump was combined with carbapenemase in 1% of P. aeruginosa and all *A. baumannii*.

According to reports of other researchers, NDM-1-producing bacterium is one of the threatening isolates, while we found that 9% of CRAB pathogens were NDM-1 positive. Unfortunately, these results should be disturbing for Iran’s health system. Obtained results show that OXA-51 and OXA-23 are the most prevalent carbapenemase in isolated *A. baumannii*, as confirmed by other studies (5, 17, 32). Another responsible resistance mechanism is the efflux pump. According to real-time PCR results from evaluating AdeB gene expression, 29% of CRAB pathogens use the efflux pump mechanism. In previously published studies, several researcher groups worked on CRAB’s efflux pump mechanism and reported similar results to the findings of the current study (33-35).

## Conclusion

The existence of different antibiotic-resistant mechanisms of *P. aeruginosa* and *A. baumannii* can cause cross antibiotic resistance, lead to the appearance of MDR and/or strains, and make the treatment difficult. The increasing number of NDM-1-producing bacteria is a very serious problem to combat in terms of antibiotic resistance. Therefore, finding a way to inhibit efflux pumps is quite essential for controlling the cross-resistance and appearance of MDR strains of bacteria.
